# Energy-dense versus routine enteral nutrition in critically ill patients: a systematic review and meta-analysis

**DOI:** 10.3389/fnut.2025.1645211

**Published:** 2025-09-01

**Authors:** Zonghong Zhang, Chuanlai Zhang, Huiling Pan, Ruiqi Yang, Yin Fang

**Affiliations:** ^1^Intensive Care Unit, The Second Affiliated Hospital of Chongqing Medical University, Chongqing, China; ^2^School of Nursing, Chongqing Medical University, Chongqing, China

**Keywords:** energy-dense, enteral nutrition, critically ill patients, routine, systematic review

## Abstract

**Background and aim:**

Critically ill patients often experience low target attainment rates with enteral nutrition (EN), leading to malnutrition and poor clinical outcomes. Energy-dense EN may improve caloric delivery and reduce the risk of malnutrition. However, its effects on other clinical outcomes remain unclear. This systematic review aimed to evaluate the impact of energy-dense EN in critically ill patients.

**Methods:**

A systematic search was conducted in PubMed, Embase, Web of Science, Cochrane Library, Clinical Trials, China Knowledge Network Infrastructure (CNKI), Wanfang Data, and Weipu databases from inception to December 2024. Two researchers independently screened studies and extracted data. Randomized controlled trials (RCTs) comparing energy-dense EN with routine EN in critically ill patients were included. Outcomes assessed included diarrhea, gastric residual volume (GRV), vomiting or reflux, mortality, total hospital length of stay (LOS), intensive care unit (ICU) LOS, duration of mechanical ventilation, and nutritional status. The risk of bias was assessed using the Cochrane RoB 2.0 tool. Meta-analyses were performed using Review Manager (RevMan), and the quality of evidence was evaluated using the Grading of Recommendations Assessment, Development and Evaluation (GRADE) approach.

**Results:**

A total of 380 studies were identified, and 10 RCTs comprising 4,473 patients were included. Compared with routine EN, energy-dense EN significantly reduced the duration of mechanical ventilation (MD = −37.41, 95% CI: −60.57 to −14.25, *I*^2^ = 75%) and ICU LOS (MD = −1.24, 95% CI: −1.49 to −0.99, *I*^2^ = 17%). Nutritional indicators such as albumin (MD = 4.92, 95% CI: 2.69–7.16, *I*^2^ = 89%) and prealbumin (MD = 55.97, 95% CI: 39.04–72.90, *I*^2^ = 86%) were significantly improved. However, there were no significant differences in total hospital LOS, mortality, or gastrointestinal complications such as diarrhea and vomiting/reflux. A slight increase in the risk of high GRV was observed (relative risk (RR) = 1.28, 95% CI: 1.19–1.37, *I*^2^ = 2%).

**Conclusion:**

Energy-dense EN appears to be safe and effective for critically ill patients, with benefits in nutritional status and reductions in ICU LOS and mechanical ventilation duration. However, this study has limitations, including potential bias in the included RCTs and inconsistent definitions of GRV. Future large-scale, high-quality, and multicenter RCTs with rigorous methodology are needed to validate these findings.

**Systematic review registration:**

https://www.crd.york.ac.uk/PROSPERO/recorddashboard.

## 1 Introduction

Critically ill patients in the intensive care unit (ICU) are in a hypercatabolic and hypermetabolic state during the acute phase of illness. Combined with the inability to maintain oral intake due to medical treatments such as pharmacotherapy and mechanical ventilation, these patients are particularly susceptible to rapid development of severe malnutrition (1). Studies have shown that the incidence of malnutrition in patients with critical illness ranges from 38 to 78% (2). Malnutrition can lead to complications such as acquired muscle weakness and infections, significantly affecting patient prognosis (3) and even increasing mortality rates (4). Therefore, early nutritional intervention is essential to reduce the incidence of malnutrition. Guidelines (5) strongly recommend initiating nutritional therapy within 24–48 h of ICU admission for hemodynamically stable patients, with particular emphasis on enteral nutrition (EN). EN offers irreplaceable benefits, including cost-effectiveness, alignment with normal physiology, and the ability to protect the gastrointestinal mucosal barrier (6, 7), among others. However, EN delivery in ICU settings is frequently interrupted due to various factors, such as diagnostic procedures, fluid restriction protocols, surgical interventions, and feeding intolerance, resulting in failure to achieve the prescribed caloric target (8, 9). A recent large observational study found that only 17.8% of patients achieved 80% of their target energy intake through EN by the seventh day of hospitalization (10). This persistent caloric deficit represents a major contributing factor to malnutrition in critically ill patients, underscoring the urgent need for optimized nutritional support strategies.

Supplemental parenteral nutrition (PN) can improve target calorie attainment to some extent; however, there is ongoing clinical debate regarding the early initiation of PN (11). Furthermore, early implementation of PN may increase the risk of infection, economic burden, and even the duration of ICU stay (12, 13). Therefore, increasing the actual EN intake in critically ill patients is particularly important. The primary strategies to enhance caloric delivery via EN include increasing the feeding rate, initiating EN early, or using energy-dense EN formulas. Previous meta-analyses (14) have mainly focused on comparing the gastrointestinal complications associated with different caloric intakes during early EN in critically ill patients, while overlooking the potential differences in clinical outcomes resulting from various strategies used to increase EN intake.

In recent years, an increasing number of studies have shown that compared to routine EN, energy-dense EN provides higher caloric intake per unit volume, with a greater proportion of fat and protein. This composition is more favorable for reducing muscle loss, improving nutritional support, lowering the incidence of malnutrition, and enhancing the nutritional status of critically ill patients (15, 16). It is also particularly beneficial for ICU patients who require volume-restricted feeding. A large RCT (17) indicated that energy-dense EN is relatively safe but may increase the risk of high gastric residual volume (GRV). However, recent studies have reported contrasting findings. Moreover, the impact of energy-dense EN on hospital length of stay, duration of mechanical ventilation, and complications such as infections has yielded inconsistent results across previous studies, making it difficult to draw definitive conclusions (17–20). Therefore, this systematic review and meta-analysis aim to evaluate the efficacy and safety of energy-dense EN in critically ill patients, clarify its clinical effects, and provide reliable evidence to inform enteral nutrition practices in critical care settings.

## 2 Methods

This study was conducted and reported according to the Preferred Reporting Items for Systematic Reviews and Meta-Analyses (PRISMA) statement (21). Additionally, this study has been registered in the International Prospective Register of Systematic Reviews (PROSPERO) with registration number CRD42024621257.

### 2.1 Search strategy

In this study, a literature search was conducted in PubMed, Embase, Web of Science, Cochrane Library, Clinical Trials, China Knowledge Network Infrastructure (CNKI), Wanfang Data, and Weipu databases, covering publications from the inception of each database through December 2024. The search was conducted using a combination of subject terms, free terms, keywords, and Boolean operators. The following terms were used: “Intensive Care Units” AND “High-Energy OR Energy-Dense OR High-Energy-Density” AND “Enteral Nutrition.” Additionally, references of the included studies were manually searched to identify relevant studies. The specific search strategy is provided in Supplementary file S1.

### 2.2 Selection criteria

The inclusion criteria were determined by following the PICOS framework: (1) Participants: critically ill adult patients (aged ≥18 years); (2) Intervention: experimental group receiving energy-dense EN (EN formulations = 1.5 kcaL/mL); (3) Control: control group receiving routine EN (EN formulations = 1 kcaL/mL); (4) Outcomes: studies must report at least one primary or secondary outcome [Primary outcomes: 1. diarrhea, 2. high gastric residual volume, and 3. vomiting or regurgitation; Secondary outcomes: 1. mortality, 2. total length of hospital stay, 3. ICU length of stay, 4. Duration of mechanical ventilation, and 5. nutritional status-related indicators (e.g., albumin and prealbumin)]; (5) Study design: Randomized controlled trials (RCTs).

We excluded the following studies: (1) duplicate publications; (2) editorials, conference abstracts, study protocols, reviews, secondary analyses, and similar publications; and (3) studies for which the full text was not available.

### 2.3 Selection process and data extraction

The literature search was conducted by the researcher (ZZH) and imported into NoteExpress version 4.1.0.10030 software, with duplicate articles removed. Two researchers independently reviewed the titles and abstracts based on the inclusion and exclusion criteria to exclude studies that did not meet the standards, followed by a full-text review to select eligible studies. Additionally, the reference lists of the included studies were examined to identify other potential studies. In case of disagreements, discussions between the two researchers were held, and a third researcher (ZCL) was consulted if necessary to reach a consensus. Based on the final selection of studies, the two authors independently extracted data using a pre-designed Excel spreadsheet, which included information such as the first author, publication year, country, sample size, feeding protocol, blinding status, primary outcomes, and secondary outcomes. Any inconsistencies in data extraction were resolved through discussion between the two researchers to reach a consensus.

### 2.4 Risk of bias assessment

Two researchers (ZZH and PHL) independently assessed the risk of bias in the randomized studies using the Cochrane Risk-of-Bias Tool for Randomized Trials version 2 (RoB 2) (22). The risk of bias was categorized into three levels: low risk, some concerns, and high risk. The bias assessment covered five domains: randomization process, deviations from intended interventions, missing outcome data, measurement of outcomes, and selection of reported results. Any disagreements were resolved through discussion between the two researchers, and if necessary, a third researcher (ZCL) was consulted to reach a consensus. Additionally, the overall quality of evidence was evaluated using the Grading of Recommendations Assessment, Development and Evaluation (GRADE) approach (23), considering five factors: study limitations, inconsistency of results, indirectness, imprecision, and reporting bias.

### 2.5 Data analysis

In this study, extracted data will be analyzed using Review Manager version 5.3 (RevMan, The Cochrane Collaboration, Oxford, UK) to calculate relative risks (RR) and 95% confidence intervals (CI) for dichotomous variables. For continuous variables, the mean difference (MD) and 95% CI will be estimated as the effect measure. A fixed-effects model will be used if the index of inconsistency (*I*^2^ < 50%) and the chi-squared test (*p* ≥ 0.10) indicate no significant heterogeneity. If significant heterogeneity is present (*p* < 0.10 and *I*^2^ ≥ 50%), a random-effects model will be applied. For continuous variables reported as median (interquartile range), the means and standard deviations will be calculated using the formula (24) based on the sample size, followed by meta-analysis. Sensitivity analysis will be conducted to assess the stability and reliability of the results. Additionally, funnel plot analysis will be performed for the final included studies, and Egger’s test (*p* < 0.05 in a two-sided test) will be used to assess reporting bias.

## 3 Results

### 3.1 Study selection process

**Figure 1:**
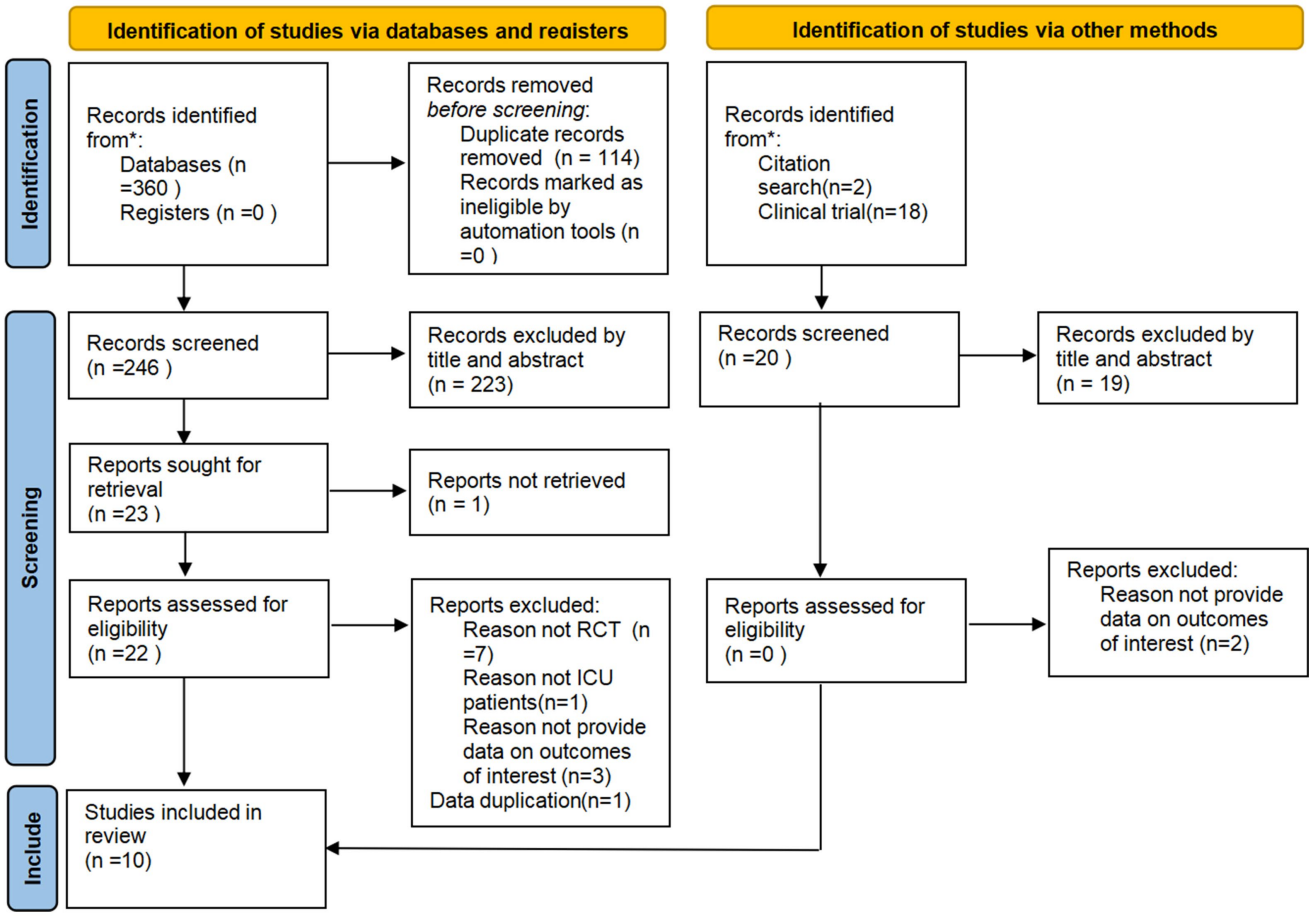
Study selection.

A total of 378 articles were retrieved from seven databases. After removing duplicates using the NoteExpress software, 264 articles remained. The researcher then screened the studies, ultimately including 10 studies. All included studies were RCTs, and the detailed screening process is shown in Figure 1.

### 3.2 Study characteristics

A total of 10 studies were included in this research, all of which were RCTs conducted between 2013 and 2023. The studies involved 4,473 participants, with sample sizes ranging from 20 to 1,941, from three countries: China (25–31) (*n* = 7), Australia (17, 19) (*n* = 2), and Russia (19) (*n* = 1). The 10 studies included critically ill patients with conditions such as severe tuberculosis, severe burn, severe traumatic brain injury, severe pneumonia, and other serious illnesses. EN was administered within 24–48 h of onset in all studies. Table 1 presents the basic characteristics of the included studies.

**Table 1:** Characteristics of included studies in chronological order.

Author, Year	Country	Patient population	Energy-dense EN formula kcal/ml	Routine EN formula kcal/ml	Targeted energy attainment program
Hao 2009 (26)	China	Critically ill burn patients	1.5 kcal/mL (enteral nutrition emulsion total parenteral – high energy (TP-HE)) *N* = 20	1 kcal/mL (regular diet TP-HE) *N* = 20	500 mL on day 1. If there were no adverse effects, such as regurgitation, the dosage was increased to 1,000 mL on day 2 and then to 1,500 mL on day 3 and maintained.
Li 2013 (27)	China	Patients with severe traumatic brain injury	1.5 kcal/mL (enteral nutrition emulsion (TP-HE)) *N* = 30	1 kcal /mL (enteral nutritional suspension (total protein formula (TPF))) *N* = 30	The first 24 h provided 40% of the energy requirement, which was increased by 30% every 24 h until the target dose was reached.
Peak 2014 (20)	Australia	Critically ill patients	1.5 kcaL/mL (Fresubin 2,250 Complete) *N* = 57	1 kcal/mL (Fresubin 2,250 Complete) *N* = 55	The study enteral nutrition was delivered at a goal rate of 1 mL kcal/(kg-h)
Mai 2015 (28)	China	Critical tuberculosis patients	1.5 kcal/mL (enteral nutrition emulsion (TP-HE)) *N* = 32	1 kcal/mL (enteral nutritional suspension (TPF)) *N* = 32	Extrapolation from the formula, Harris–Bendict
Efremov 2017 (19)	Russia	Critically ill cardiac patients	1.3 kcaL/mL *N* = 20	1 kcal /ml *N* = 20	Not mentioned
Chapman 2018 (17)	Australia	Critically ill patients	1.5 kcal/ml (Fresubin Energy Fiber Tube Feed) *N* = 1,935	1.0 kcaL/mL (Fresubin 1,000 Complete Tube Feed) *N* = 1,941	The target rate groups were 1 mL kcal/(kg-h)
Fan 2020 (30)	China	Patients with severe traumatic brain injury	1.5 kcal/mL (whole protein enteral total nutrition emulsion) *N* = 21	1 kcal/mL (whole protein enteral total nutrition emulsion) *N* = 21	Energy requirements of 22 ~ 25 kcal/(kg-d)
Tao 2021 (31)	China	Patients with severe traumatic brain injury	1.5 kcal/mL (whole protein enteral total nutrition emulsion) *N* = 27	1 kcal/mL (whole protein enteral total nutrition emulsion) *N* = 27	Energy requirements of 22 ~ 25 kcal/(kg-d)
Yan 2022 (25)	China	Critical pneumonia patient	1.5 kcal/ml *N* = 45	1 kcal/mL *N* = 44	500 mL on day 1. If there were no adverse effects such as regurgitation, the dosage was increased to 1,000 mL on day 2 and then to 1,500 mL on day 3 and maintained
Li 2023 (29)	China	Patients with severe traumatic brain injury	1.5 kcal/mL *N* = 48	1 kcal/mL *N* = 48	Not mentioned

### 3.3 Risk of bias

**Figure 2:**
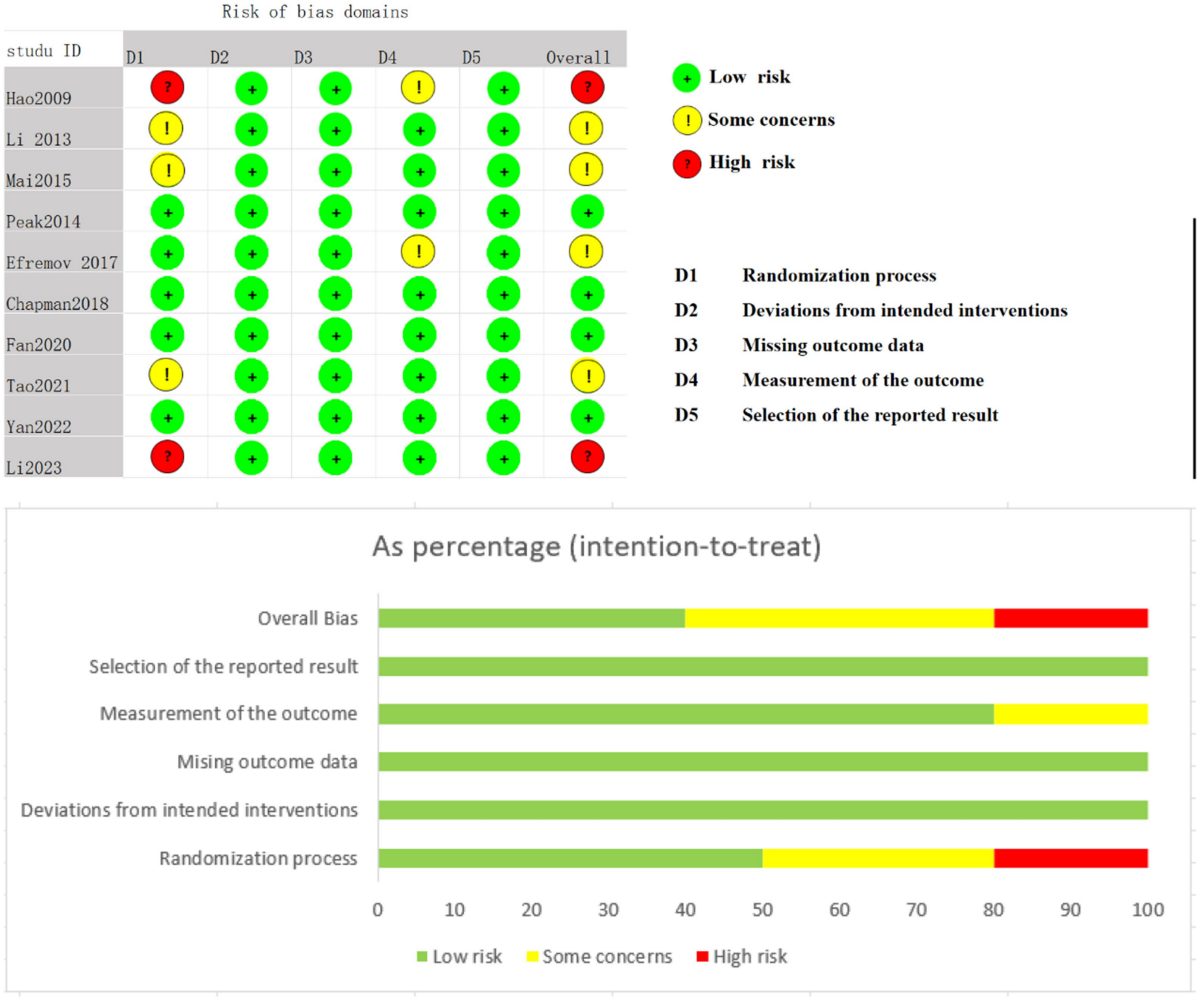
Evaluation of risk of bias.

Four studies were assessed as having a low risk of bias, four studies raised some concerns, and two studies were classified as having a high risk of bias, primarily due to issues in the randomization process. Both high-risk studies lacked random sequence generation and allocation concealment. Four studies raised concerns because allocation concealment was not performed, leading to potential biases. This can be seen in detail in Figure 2.

## 4 Outcome

### 4.1 Primary outcomes

#### 4.1.1 Diarrhea

**Figure 3:**
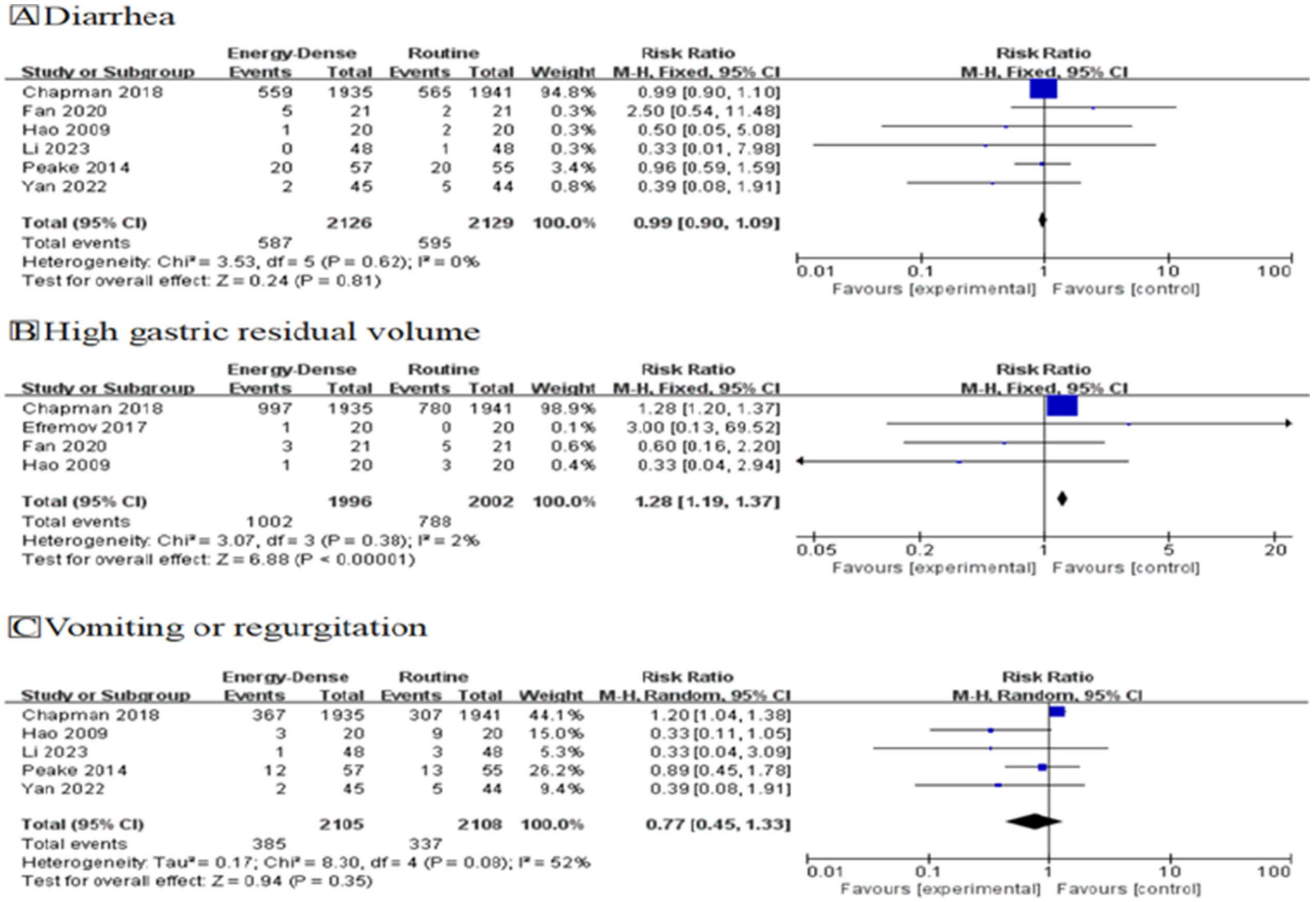
Forest plots of primary outcomes, **(A)** Diarrhea, **(B)** High gastric residual volume, **(C)** Vomiting or regurgitation.

A total of 6 studies (17, 20, 25, 26, 29, 30), including 4,255 critically ill patients (2126 in the experimental group and 2129 in the control group), provided usable data for the meta-analysis. Pooled analysis showed no significant difference in the incidence of diarrhea between the two groups (RR = 0.99, 95% CI: 0.90–1.09, Figure 3A). No heterogeneity was observed between the studies (*I^2^* = 0%). However, due to the inclusion of two studies with a high risk of bias, the evidence was downgraded one level for bias risk. According to GRADE ratings, the overall quality of the evidence was assessed as moderate.

#### 4.1.2 High gastric residual volume

A total of 4 studies (17, 19, 26, 30), including 3,998 critically ill patients (1,996 in the experimental group and 2,002 in the control group), provided usable data for the meta-analysis. Pooled analysis showed that critically ill patients fed energy-dense EN formulas were more likely to experience gastric residuals compared to controls (RR = 1.28, 95% CI: 1.19–1.37, Figure 3B). The studies showed minimal heterogeneity (*I*^2^ = 2%). Due to the inclusion of one study with a high risk of bias, the evidence was downgraded one level for bias risk. According to GRADE ratings, the overall quality of the evidence was assessed as moderate.

#### 4.1.3 Vomiting or regurgitation

A total of 5 studies (17, 20, 25, 26, 29), including 4,213 critically ill patients (2,105 in the experimental group and 2,108 in the control group), provided usable data for the meta-analysis. Pooled analysis showed no significant difference in the incidence of vomiting or regurgitation between the two groups (RR = 0.77, 95% CI: 0.45–1.33, Figure 3C). There was considerable heterogeneity between the studies (*I*^2^ = 52%). Due to the inclusion of two studies with a high risk of bias, the evidence was downgraded one level for risk of bias. Additionally, because the *I^2^* value was greater than 50%, the evidence was further downgraded one level for inconsistency. According to GRADE ratings, the overall quality of the evidence was assessed as low.

### 4.2 Secondary outcomes

#### 4.2.1 Mortality

**Figure 4:**
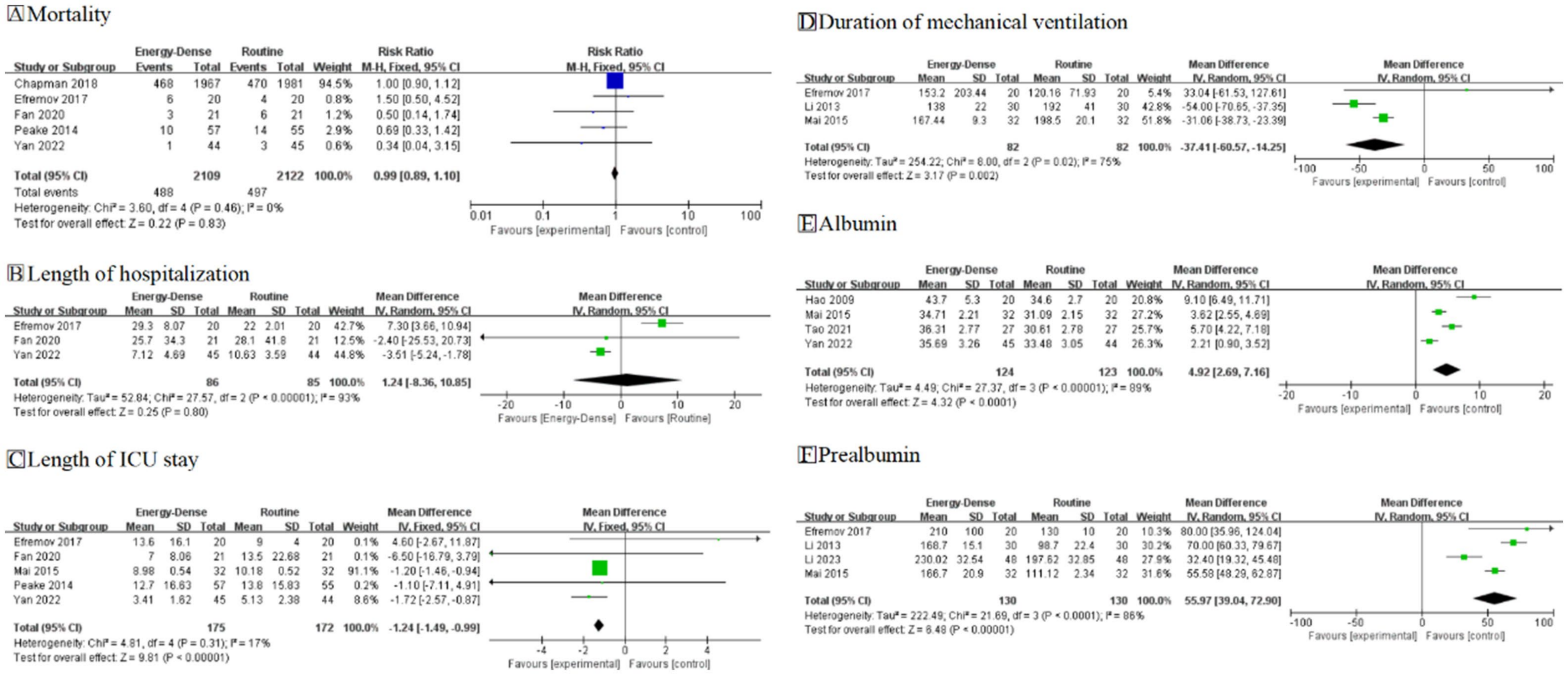
Forest plots of secondary outcomes. **(A)** Mortality; **(B)** Total length of hospitalization; **(C)** Length of ICU stay; **(D)** Duration of mechanical ventilation; **(E)** Albumin; **(F)** Prealbumin.

A total of 5 studies (17, 19, 20, 25, 30), including 4,231 critically ill patients (2,109 in the experimental group and 2,122 in the control group), provided usable data for the meta-analysis. The pooled analysis revealed no statistically significant difference in mortality between the two groups (RR = 0.99, 95% CI: 0.89–1.10, Figure 4A). No heterogeneity was observed across the studies (*I^2^* = 0%). No bias was detected. According to GRADE ratings, the overall quality of the evidence was assessed as high.

#### 4.2.2 Total length of hospitalization

A total of 3 studies (19, 25, 30), including 171 critically ill patients (86 in the experimental group and 85 in the control group), provided usable data for the meta-analysis. The pooled analysis showed no statistically significant difference in total hospitalization time (MD = 1.24, 95% CI: −8.36 to 10.85, Figure 4B). However, substantial heterogeneity was observed between the studies (*I*^2^ = 93%). Due to this large heterogeneity, the evidence was downgraded one level for inconsistency. Additionally, because of the small sample size, the evidence was downgraded one level for uncertainty. According to GRADE ratings, the overall quality of the evidence was assessed as low.

#### 4.2.3 Length of ICU stay

A total of five studies (19, 20, 25, 28, 30), including 347 critically ill patients (175 in the experimental group and 172 in the control group), provided usable data for the meta-analysis. The pooled analysis demonstrated that critically ill patients fed energy-dense EN formulas had a reduced ICU length of stay compared to controls (MD = −1.24, 95% CI: −1.49 to −0.99, Figure 4C). Little heterogeneity was noted between the studies (*I*^2^ = 17%). Due to the small sample size, the evidence was downgraded one level for uncertainty. According to GRADE ratings, the overall quality of the evidence was assessed as moderate.

#### 4.2.4 Duration of mechanical ventilation

A total of 3 studies, comprising 164 critically ill patients (82 in the experimental group and 82 in the control group), provided usable data for the meta-analysis. The pooled analysis indicated that energy-dense EN feeding reduced the duration of mechanical ventilation compared to the control group (MD = −37.41, 95% CI = −60.57 to −14.25, Figure 4D). However, substantial heterogeneity was observed among the study results (*I^2^* = 75%). Although heterogeneity was high, the 95% confidence intervals were consistently in the same direction, suggesting no major inconsistency. Due to the limited sample size, the certainty of evidence was downgraded by one level. According to the GRADE rating, the overall evidence quality was assessed as moderate.

#### 4.2.5 Nutrition-related indicators

A total of 4 studies, including 247 critically ill patients (124 in the experimental group and 123 in the control group), reported post-feeding albumin outcomes. Energy-dense EN was more effective in improving patients’ nutritional status compared to routine EN (MD = 4.92, 95% CI: 2.69–7.16, *I^2^* = 89%, Figure 4E). Due to the small number of patients included in the studies, the certainty of the evidence was downgraded by one level. According to the GRADE rating, the overall quality of the evidence was assessed as moderate. Similarly, four studies, including 260 critically ill patients (130 in the experimental group and 130 in the control group), reported post-feeding prealbumin outcomes. Consistent with the albumin results, energy-dense EN demonstrated a greater improvement in patients’ nutritional status (MD = 55.97, 95% CI: 39.04–72.90, *I*^2^ = 86%, Figure 4F). Although the heterogeneity in I^2^ was high, the 95% CI was in the same direction, indicating no significant inconsistency. Due to the small number of patients included in the studies, the certainty of the evidence was downgraded by one level, and the overall quality of the evidence was assessed as moderate according to the GRADE rating.

### 4.3 Qualitative analysis

#### 4.3.1 Infection

A total of five studies reported (25, 27, 29–31) the incidence of infectious complications. Due to variations in the definitions of infectious complications across these studies, a meta-analysis could not be performed. Three studies indicated that energy-dense EN reduced the incidence of infectious complications in critically ill patients (25, 27, 30). However, two studies found no significant difference in infection rates between energy-dense and routine EN, with one study assessing infection based on the rate of positive blood cultures (29, 31).

#### 4.3.2 Immune indicators

A total of five studies reported (25–29) immune function-related indicators in critically ill patients. Three studies suggested that energy-dense EN was more effective than routine EN in improving immune function (25, 28, 29). It was associated with lower C-reactive protein (CRP) levels, increased production of immunoglobulin (IgA), IgG antibodies, and other immunoglobulins, and improved cellular immune function indicators, such as the cluster of differentiation 3 (CD3^+^), CD4^+^, and CD8^+^ T cells, as well as the CD4^+^/CD8^+^ T cell ratio. However, two studies comparing total lymphocyte counts found no significant difference between energy-dense and routine EN (26, 27).

## 5 Sensitivity analysis and publication bias

A sensitivity analysis was conducted by comparing the results from the combined fixed-effects and random-effects models. Meta-analysis was used to combine the study results, and the RR values along with their confidence intervals from the two models were compared. No significant differences were observed, indicating that the combined results were stable and less sensitive to the choice of model. The specific results are presented in Table 2. Additionally, since there were no studies with more than 10 items in the pooled results of this study, funnel plot analysis was not performed.

**Table 2:** Sensitivity analysis form.

Outcome indicator	Effect model	Effect size	95% CI	Effect model	Effect size	95 CI%
Diarrhea	FEM	RR = 0.99	(0.90,1.09)	REM	RR = 0.99	(0.9, 1.09)
Gastric residual volume	FEM	RR = 1.28	(1.19,1.37)	REM	RR = 1.25	(1.03, 1.52)
Vomiting or regurgitation	FEM	RR = 1.41	(1.00, 1.31)	REM	RR = 0.77	(0.45, 1.33)
Mortality	FEM	RR = 0.99	(0.89, 1.10)	REM	RR = 0.99	(0.89, 1.10)
Length of hospitalization	FEM	MD = −1.52	(−3.08, 0.04)	REM	MD = 1.24	(−8.36, 10.85)
Length of ICU stay	FEM	MD = −1.24	(−1.49, −0.99)	REM	MD = −1.32	(−1.84, −0.8)
Duration of mechanical ventilation	FEM	MD = −34.71	(−41.66, −7.76)	REM	MD = −37.41	(−60.57, −14.25)
Albumin	FEM	MD = 4.07	(3,38, 4.77)	REM	MD = 4.92	(2.69, 7.16)
Prealbumin	FEM	MD = 56.46	(51.18, 61.73)	REM	MD = 55.97	(39.04, 72.90)

FEM = fixed effects model; REM = random effects model.

## 6 Discussion

Through a systematic review and meta-analysis of 10 RCTs involving 4,473 patients, we found that energy-dense EN reduced the duration of mechanical ventilation and ICU stay without increasing the risk of gastrointestinal complications such as diarrhea, vomiting, and regurgitation. Additionally, it significantly improved patients’ nutritional status compared to routine feeding. However, no significant differences were observed in mortality or total hospital stay, and there was a potential increase in the risk of high gastric residual volume. Therefore, the application of energy-dense EN in critically ill patients can enhance target caloric achievement, improve nutritional status, accelerate recovery, and help alleviate the financial burden on patients.

Regarding gastrointestinal complications, the risks of diarrhea, vomiting, and reflux were similar between energy-dense and routine EN formulas. However, energy-dense feeding may increase the risk of high gastric residual volumes to some extent. Contrary to the initial hypothesis, although energy-dense formulas have higher osmolality than standard formulas, they did not increase the risk of osmotic diarrhea. In addition, this study found that compared with the control group, energy-dense EN did not increase the risk of vomiting and reflux. This finding is consistent with previous research and may be related to the reduced feeding volume required for energy-dense formulas. Nevertheless, energy-dense EN may modestly elevate the risk of high gastric residual volumes in critically ill patients when compared with routine EN, consistent with the findings of a 2020 observational study (32). Energy-dense formulas often contain a higher proportion of fat. Among macronutrients, lipids exhibit the slowest gastric emptying rate compared with carbohydrates and proteins (33). Moreover, the increased energy density may enhance the interaction between nutrients and small intestinal mucosal receptors, which reinforces the inhibitory effect on gastric emptying (34). These factors collectively contribute to the elevated risk of high gastric residual volumes. It is worth noting that the study by Chapman et al. (17) had a large sample size of 3,876 patients, accounting for 98.9% of the pooled data in the meta-analysis. Their population included critically ill patients with various disease types, including those with gastrointestinal conditions, who were already at elevated risk for high gastric residuals—potentially introducing bias into the results. Additionally, the definition of high gastric residual volume varies across countries, and differences in the formulations used for EN may have further contributed to heterogeneity. Future research should aim to standardize EN compositions and the diagnostic criteria for high gastric residuals, enabling more consistent assessments. In conclusion, while energy-dense EN may be associated with a higher risk of elevated gastric residual volumes, it does not appear to increase the incidence of other gastrointestinal adverse effects, suggesting an acceptable safety profile.

Compared with routine EN formulas, energy-dense EN significantly shortened the duration of mechanical ventilation and ICU stay in critically ill patients and improved their nutritional status. However, no significant differences were observed in overall hospital length of stay or mortality. The reduction in mechanical ventilation time associated with energy-dense EN may be explained by two factors. First, these formulas typically contain a lower proportion of carbohydrates, which may reduce CO₂ production and thereby improve oxygenation in some patients with respiratory distress (35). Second, energy-dense EN helps critically ill patients achieve adequate nutritional targets more quickly, which can mitigate muscle loss, increase the likelihood of successful weaning, and ultimately shorten the duration of mechanical ventilation (36). Additionally, energy-dense EN requires a smaller feeding volume and less time to achieve energy targets, thereby ensuring adequate nutritional support and promoting recovery during the rehabilitation phase. This facilitates ICU discharge and reduces ICU length of stay (37).

Serum albumin and prealbumin are indicators that reflect protein-energy malnutrition (38). Our analysis showed that energy-dense EN resulted in greater increases in these markers, indicating better improvement in nutritional status among critically ill patients, consistent with the findings of Pardo et al. (39). Energy-dense EN may also enhance immune function to some extent; however, due to inconsistent reporting of infection-related outcomes across the included studies, a meta-analysis could not be conducted on infectious complications, and the effect of energy-dense EN on infection prevention remains unclear. In line with previous retrospective studies (37, 40), no significant differences were observed in mortality or total hospital stay among critically ill patients. Clinical outcomes in this population are influenced by multiple factors, and the composition of EN formulas may have a limited effect on mortality. Moreover, the limited number of included studies may not provide sufficient statistical power to detect potential differences in mortality associated with energy-dense EN. Therefore, further high-quality studies are needed to clarify the impact of EN with different energy densities on mortality and infectious complications in critically ill patients. In summary, the findings suggest that energy-dense EN offers certain clinical benefits when applied to critically ill patients.

## 7 Strengths and limitations

To the best of our knowledge, this is the first systematic review and meta-analysis to evaluate the safety and efficacy of energy-dense EN formulas in critically ill patients. The results of the meta-analysis indicate that energy-dense EN is relatively safe for use in this population, and it can help reduce ICU length of stay, shorten the duration of mechanical ventilation, and improve patients’ nutritional status. However, several limitations should be noted. First, this study only included literature published in Chinese and English, with 7 out of the 10 included RCTs published in Chinese. This may introduce a degree of selection bias. Second, the overall methodological quality of the included RCTs was moderate. Two studies were assessed as having a high risk of bias, and five had some concerns, mainly due to the lack of allocation concealment, which may limit the strength and generalizability of the evidence. Third, due to the limited focus of current research on the impact of different energy-dense EN formulas on critically ill patients, the number of included studies was relatively small. In addition, critically ill patients with different conditions, such as severe burns or post-cardiac surgery, may have varying durations of hospital stay and mechanical ventilation, which contributed to the observed heterogeneity in outcomes such as mechanical ventilation duration and total hospital stay. The small number of studies also limited the ability to perform subgroup analyses. Future studies with more rigorous designs are needed to explore the differential effects of energy-dense EN in critically ill patients with different diseases. Moreover, the threshold for defining high gastric residual volume varies across countries and clinical guidelines, which may further increase heterogeneity in outcome measures. Therefore, future research should involve multicenter international collaboration, standardization of feeding protocols and evaluation methods, and unified definitions of outcome indicators. Well-designed randomized controlled trials are needed to provide more robust evidence for clinical practice.

## 8 Conclusion

In summary, energy-dense EN can shorten the duration of mechanical ventilation and ICU stay compared to routine EN. However, there is no significant difference in total hospitalization time, mortality, or gastrointestinal complications, such as diarrhea, vomiting, and regurgitation, though it may increase the risk of high gastric residual volume. Additionally, energy-dense EN effectively improves nutritional status indicators, such as prealbumin, in critically ill patients. Despite the limitations of this study, the evidence suggests that energy-dense EN is both safe and effective for critically ill patients. In the future, more comprehensive and rigorous RCTs are needed to further explore its effects.

## Data Availability

Publicly available datasets were analyzed in this study. This data can be found here: no.
