# Supplementary material for: Energy-dense versus routine enteral nutrition in critically ill patients: a systematic review and meta-analysis
Source: Front Nutr. 2025 Sep 1;12:1645211. doi: 10.3389/fnut.2025.1645211 (PMC12434126; doi:10.3389/fnut.2025.1645211)
Supplement: Supplementary file 1 [file Table_1.docx]

Pumed

| #1 | "High-Calorie"[Title/Abstract] OR "Calorically-Dense"[Title/Abstract]OR"Energy-Rich"[Title/Abstract] OR "High-Energy"[Title/Abstract] OR "Nutrient-Dense"[Title/Abstract] OR "Energy-Concentrated"[Title/Abstract] OR "Caloric-Dense"[Title/Abstract] OR "Concentrated"[Title/Abstract] OR "Fortified"[Title/Abstract] OR "Energy-Enhanced"[Title/Abstract] OR "High-Energy-Density"[Title/Abstract] OR "Energy-Dense"[Title/Abstract] OR "High-Protein, High-Energy Enteral"[Title/Abstract])) OR "High-Energy Enteral,High-Protein"[Title/Abstract] OR "Energy Density"[Title/Abstract] OR "Calorie-Rich"[Title/Abstract] |
| --- | --- |
| #2 | "Enteral Nutrition"[Mesh] OR"Nutritional Supplement"[Title/Abstract] OR "Enteral Formula[Title/Abstract]" OR "Enteral Feeding"[Title/Abstract] OR "Nutritional Support"[Title/Abstract] OR "Enteral Formulations"[Title/Abstract] OR "Enteral Formulas"[Title/Abstract] OR "Feeding, Tube"[Title/Abstract] OR "Enteral Feeding"[Title/Abstract] OR "Gastric Feeding Tubes"[Title/Abstract] OR "Tube, Gastric Feeding"[Title/Abstract] OR "Feeding, Force[Title/Abstract]" OR "Force Feedings"[Title/Abstract] OR "Feedings, Force"[Title/Abstract] OR "Tube Feeding"[Title/Abstract] OR "Nutrition, Enteral"[Title/Abstract] |
| #3 | **"Intensive Care Units"[Mesh]) OR "Respiration, Artificial"[Title/Abstract] OR "Ventilators, Mechanical"[Title/Abstract] OR "intensive care"[Title/Abstract] OR "intensive care"[Title/Abstract] OR "Critical Illness"[Title/Abstract] OR "critical care"[Title/Abstract] OR "critically ill"[Title/Abstract]))** |
| #4 | #1 AND #2 AND #3 |

Cochrane

| #1 | （High-Calorie）:ab,ti，kw or （Calorically-Dense）:ab,ti，kw or （High-Energy）:ab,ti，kw or （Energy-Rich）:ab,ti，kw or （Energy-Concentrated）:ab,ti，kw or （Nutrient-Dense）:ab,ti，kw or （Caloric-Dense）:ab,ti，kw or （Concentrated）:ab,ti，kw or （Fortified ）:ab,ti，kw or （Energy-Enhanced）:ab,ti，kw or （High-Energy-Density）:ab,ti，kw or （Energy-Dense）:ab,ti，kw or （High-Protein, High-Energy Enteral）:ab,ti，kw or （High-Energy Enteral，High-Protein）:ab,ti，kw or （ Energy Density）:ab,ti，kw or （Calorie-Rich）:ab,ti，kw |
| --- | --- |
| #2 | "Enteral Nutrition"[Mesh] |
| #3 | （intensive care）:ab,ti，kw or （Critical Illness）:ab,ti，kw or （critically ill）:ab,ti，kw or （critical care）:ab,ti,kw |
| #4 | #1 AND #2 AND #3 |

Web of Science

| #1 | (((((((((((((((TI=(High-Calorie)) OR TI=(Calorically-Dense)) OR TI=(Energy-Rich)) OR TI=(High-Energy)) OR TI=(Nutrient-Dense)) OR TI=(Energy-Concentrated)) OR TI=(Caloric-Dense)) OR TI=(Concentrated)) OR TI=(Fortified )) OR TI=(Energy-Enhanced)) OR TI=(High-Energy-Density)) OR TI=(Energy-Dense)) OR TI=(High-Protein, High-Energy Enteral)) OR TI=(High-Energy Enteral，High-Protein)) OR TI=( Energy Density)) OR TI=(Calorie-Rich) |
| --- | --- |
| #2 | (((((((((((((((AB=(High-Calorie)) OR AB=(Calorically-Dense)) OR AB=(Energy-Rich)) OR AB=(High-Energy)) OR AB=(Nutrient-Dense)) OR AB=(Energy-Concentrated)) OR AB=(Caloric-Dense)) OR AB=(Concentrated)) OR AB=(Fortified )) OR AB=(Energy-Enhanced)) OR AB=(High-Energy-Density)) OR AB=(Energy-Dense)) OR AB=(High-Protein, High-Energy Enteral)) OR AB=(High-Energy Enteral，High-Protein)) OR AB=( Energy Density)) OR AB=(Calorie-Rich)  TS=(Enteral Nutrition) |
| #3 | ((((((((((((((TI=(Nutritional Supplement)) OR TI=(Enteral Formula)) OR TI=(Enteral Feeding)) OR TI=(Nutritional Support)) OR TI=( Enteral Formulations)) OR TI=(Enteral Formulas)) OR TI=(Feeding, Tube)) OR TI=(Enteral Feeding)) OR TI=(Gastric Feeding Tubes)) OR TI=(Tube, Gastric Feeding)) OR TI=(Feeding, Force)) OR TI=(Force Feedings)) OR TI=(Feedings, Force)) OR TI=(Tube Feeding)) OR TI=(Nutrition, Enteral) |
| #4 | ((((((((((((((AB=(Nutritional Supplement)) OR AB=(Enteral Formula)) OR AB=(Enteral Feeding)) OR AB=(Nutritional Support)) OR AB=( Enteral Formulations)) OR AB=(Enteral Formulas)) OR AB=(Feeding, Tube)) OR AB=(Enteral Feeding)) OR AB=(Gastric Feeding Tubes)) OR AB=(Tube, Gastric Feeding)) OR AB=(Feeding, Force)) OR AB=(Force Feedings)) OR AB=(Feedings, Force)) OR AB=(Tube Feeding)) OR AB=(Nutrition, Enteral) |
| #5 | (((((((((((TS=(Intensive Care Units)) OR TS=(Respiration, Artificial)) OR TS=(Ventilators, Mechanical)) OR TI=(intensive care)) OR AB=(intensive care))) OR TI=(Critical Illness)) OR AB=(Critical Illness)) OR TI=(critical care)) OR AB=(critical care)) OR TI=(critically ill)) OR AB=(critically ill) |
| #6 | （#1 OR #2 ） AND （#3 OR #4) AND #5 |

Embase

| #1 | 'High-Calorie':ab,ti or 'Calorically-Dense':ab,ti or 'High-Energy':ab,ti or 'Energy-Rich':ab,ti or 'Energy-Concentrated':ab,ti or 'Nutrient-Dense':ab,ti or 'Caloric-Dense':ab,ti or 'Concentrated':ab,ti or 'Fortified ':ab,ti or 'Energy-Enhanced':ab,ti or 'High-Energy-Density':ab,ti or 'Energy-Dense':ab,ti or 'High-Protein, High-Energy Enteral':ab,ti or 'High-Energy Enteral，High-Protein':ab,ti or ' Energy Density':ab,ti or 'Calorie-Rich':ab,ti |
| --- | --- |
| #2 | 'Nutritional Supplement':ab,ti or 'Enteral Nutrition':ab,ti or 'Enteral Feeding':ab,ti or 'Enteral Formula':ab,ti or 'Enteral Formulations':ab,ti or 'Nutritional Support':ab,ti or 'Enteral Formulas':ab,ti or 'Feeding, Tube':ab,ti or 'Enteral Feeding':ab,ti or 'Gastric Feeding Tubes':ab,ti or 'Tube, Gastric Feeding':ab,ti or 'Feeding, Force':ab,ti or 'Force Feedings':ab,ti or 'Feedings, Force':ab,ti or 'Tube Feeding':ab,ti or 'Nutrition, Enteral':ab,ti |
| #3 | 'intensive care':ab,ti or 'Critical Illness':ab,ti or 'critical care':ab,ti or 'critically ill':ab,ti |
| #4 | #1 AND #2 AND #3 |

China National Knowledge Infrastructure (CNKI)

| #1 | (zhuti：changneiyingyang (jingque) OR (zhuti：guansi (jingque) OR (zhuti：changneiyingyangpeifang (jingque) OR (zhuti：changneiyingyangzhiliao (jingque) OR (zhuti：changneiyingyangzhiliao (jingque) |
| --- | --- |
| #2 | (zhuti：zhongzhengjianhubingfang (jingque) OR (zhuti：zhongzheng (jingque) OR (zhuti：ICU (jingque) OR (zhuti：icu (jingque) OR (zhuti：weizhongzheng (jingque) OR (zhuti：jixietongqi (jingque) |
| #3 | (zhuti：gaonengliangmidu (jingque) OR (zhuti：nongsuogaonengliang (jingque) OR (zhuti：nongsuo (jingque) OR (zhuti：gaoshentouya (jingque) OR (zhuti：gaochunengliang (jingque) |
| #4 | #1 AND #2 AND #3 |

Wanfang Data

| #1 | (zhuti：changneiyingyang (jingque) OR (zhuti：guansi (jingque) OR (zhuti：changneiyingyangpeifang (jingque) OR (zhuti：changneiyingyangzhiliao (jingque) OR (zhuti：changneiyingyangzhiliao (jingque) |
| --- | --- |
| #2 | (zhuti：zhongzhengjianhubingfang (jingque) OR (zhuti：zhongzheng (jingque) OR (zhuti：ICU (jingque) OR (zhuti：icu (jingque) OR (zhuti：weizhongzheng (jingque) OR (zhuti：jixietongqi (jingque) |
| #3 | (zhuti：gaonengliangmidu (jingque) OR (zhuti：nongsuogaonengliang (jingque) OR (zhuti：nongsuo (jingque) OR (zhuti：gaoshentouya (jingque) OR (zhuti：gaochunengliang (jingque) |
| #4 | #1 AND #2 AND #3 |

Weipu

| #1 | (guanjianci：changneiyingyang) OR guanjianci：guansi) OR (guanjianci：changneiyingyangpeifang) OR (guanjianci：changneiyingyangzhiliao) OR (guanjianci：changneiyingyangzhiliao) |
| --- | --- |
| #2 | (guanjianci：zhongzhengjianhubingfang) OR (guanjianci：zhongzheng) OR (guanjianci：ICU) OR (guanjianci：icu) OR (guanjianci：weizhongzheng) OR (guanjianci：jixietongqi) |
| #3 | (guanjianci：gaonengliangmidu) OR (guanjianci：nongsuogaonengliang) OR (guanjianci：nongsuo) OR (guanjianci：gaoshentouya) OR (guanjianci：gaochunengliang) |
| #4 | #1 AND #2 AND #3 |
